# HELLP Syndrome Complicated by Subcapsular Hematoma of Liver: A Case Report and Review of the Literature

**DOI:** 10.1155/2014/585672

**Published:** 2014-04-02

**Authors:** Atilla Karateke, Dilek Silfeler, Faruk Karateke, Raziye Kurt, Ayse Guler, Ismail Kartal

**Affiliations:** ^1^Department of Obstetric and Gynecology, School of Medicine, Mustafa Kemal University, 3100 Hatay, Turkey; ^2^Department of General Surgery, Numune Training and Research Hospital, 01170 Adana, Turkey; ^3^Department of Radiology, School of Medicine, Mustafa Kemal University, 3100 Hatay, Turkey

## Abstract

Subcapsular liver hematoma (SLH) is a rare complication of severe preeclampsia and HELLP syndrome. These patients must be followed up in intensive care unit for advanced medical support with infused fluid, replacement of blood products, and treatment of underlying disorders. There are a lot of therapeutic options varying from conservative management to surgical treatment including hepatic resection, hepatic artery ligation, and liver transplantation. In this report we aimed to present a 26-year-old woman with SLH secondary to HELLP syndrome.

## 1. Introduction

Subcapsular liver hematoma (SLH) has been reported in less than 2% of pregnancies complicated by HELLP syndrome (hemolysis, elevated liver enzymes, and low platelets). The incidence of SLH has been reported, 1/40.000 to 1/250.000, leading to increased rate of both maternal and perinatal morbidity and mortality [1, 2]. The symptoms of SLH may represent as epigastric, right upper quadrant or shoulder pain, abdominal distension, nausea and vomiting. SLH may result in hepatic rupture and therefore may cause life-threatening problems such as disseminated intravascular coagulation (DIC), acute liver, and kidney failure. In this paper, we reported a patient with SLH who was managed conservatively and reviewed the literature.

## 2. Case Report

A 26-year-old woman, gravida 3, parity 2, was admitted at 29 weeks of gestation with preeclampsia to our clinic. The patient's complaints were increasing headache on frontal side and visual impairment. Her past medical and family history was unremarkable. Arterial blood pressure was 170/90 mmHg and laboratory findings revealed serum aspartate aminotransaminase (AST): 350 IU/L (*N*: 5–34), serum alanine aminotransaminase (ALT): 450 IU/L (*N*: 0–55), serum lactate dehydrogenase (LDH): 480 IU/L (*N*: 125–243), serum urea and creatine to be normal, white blood cell (WBC): 13200/mm^3^ (*N*: 4000–11000), hemoglobin (Hb): 10.6 mg/dL (*N*: 11.5–16.0), and platelet count (Plt): 90.000/mm^3^ (*N*: 150.000–450000). A catheterized urine specimen demonstrated proteinuria (++).

In obstetrical ultrasonography (USG), fetal heart rate (FHR) was 140/minute and fetal measurement was compatible with 29 weeks of gestation. The patient was hospitalized for diagnosis of elevated liver enzymes and low platelets in suspicion of HELLP syndrome. Despite the fact that intravenous magnesium sulfate was given to the patient, after two hours of hospitalization, she had convulsion. USG was done by a senior obstetrician and showed FHR to be negative.

**Figure 1:**
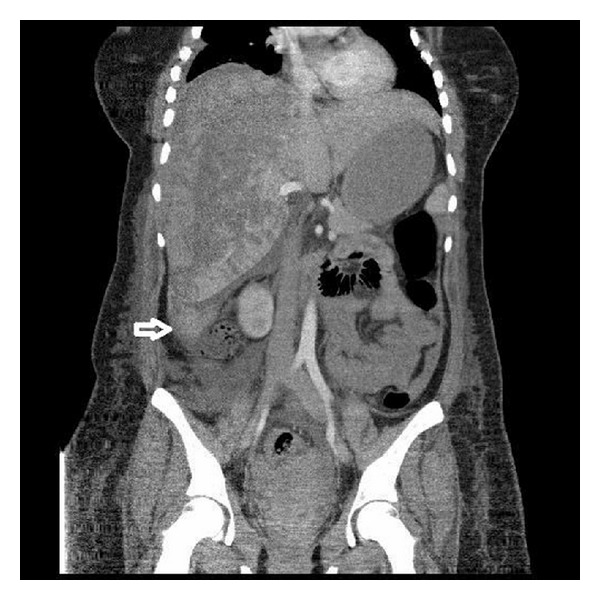
Computed abdominal tomography (CT) scan showed SLH.

After patient's approval and given necessary knowledge about intrauterine morte fetus, induction for delivery was started to the patient. An 850 gr death-born female fetus was delivered. On the second day of the postpartum period, the patient complained of severe pain on right upper quadrant and on her right shoulder. The heart rate was 120/minute and blood pressure measurement was 150/90 mmHg. On physical examination, rebound was positive and distended abdomen was noted. Laboratory results were as follows: AST: 4000 IU/L, ALT: 3450 IU/L, LDH: 980 IU/L, serum creatine (Cr): 1.8, WBC: 23200/mm^3^, Hb: 7.6 mg/dL, and Plt: 30.000/mm^3^. Transabdominal USG (TA-USG) showed a hypoechoic subcapsular mass measuring 60 × 40 mm in diameter, in anterior segment of right hepatic lobe, and also perihepatic, perisplenic, and pelvic free fluid in the abdominal cavity. After diagnosis of subcapsular liver hematoma, the patient was transferred to intensive care unit. Computed abdominal tomography (CT) scan showed SLH and was compatible with TA-USG (Figure 1). Patient was haemodynamically stable and no active intra-abdominal bleeding was observed and she was conservatively followed up in the intensive care unit. Two units of packed red blood cells and 2 units of pooled thrombocytes were transfused. High dose of corticosteroids and antihypertensive treatment had been given to the patient. Repeated ultrasound examinations were performed at a daily basis and size of hematoma was decreased at the following days. On the 9th day of the postpartum period, laboratory findings were as follows: AST: 60 IU/L, ALT: 45 IU/L, LDH: 380 IU/L, Cr: 1.2, WBC: 12000/mm^3^, Hb: 9.2 mg/dL, and Plt: 85.000/mm^3^. After the 2-week postpartum period, the patient was discharged from hospital. After the 8-week postpartum period, TA-USG was reperformed and showed no hematoma or free pelvic fluid.

## 3. Discussion

SLH in pregnancy was reported firstly by Abercombie in 1844 [3]. SLH, a rare complication of preeclampsia and HELLP, is an emergent obstetrical problem which increases the rate of severe morbidity and mortality [4]. SLH occurs in about 1-2% of all preeclampsia cases and HELLP syndrome [1, 2]. The incidence of SLH has been reported to be higher in the group of advanced maternal age and multiparous patients [5].

The underlying pathogenesis of SLH in HELLP syndrome is not well known. It has been reported that preeclamptic syndrome induces fibrin deposition, hypovolaemia, hepatic ischaemia, and infarction causing haemorrhage and SLH [6]. Thus, continuing expansion of the SLH may induce the rupture of the hepatic capsule in case of trauma such as abdominal palpation, transportation of the patient, manual removal of the placenta, uterine contractions, and vomiting which have been hypothesized as the reasons underlying this event [6]. When evaluating the SLH as histopathological, intraparenchymal haemorrhage, common microaneurysms with periportal or focal parenchymal necrosis were observed [7, 8]. On macroscopic examination of SLH, tint little bleeding areas were observed in many places under the Glisson capsule [9].

SLH in pregnancy must be followed up with hemodynamic and coagulation parameters during the management of HELLP syndrome or/and preeclampsia. TA-USG, computerized tomography (CT), and magnetic resonance imaging (MRI) can be used as diagnostic tools. Haemodynamically stable patients should be followed up conservatively by means of intensive medical support with infused fluid, replacement of blood products, and treatment of HELLP syndrome or/and preeclampsia. The administration of recombinant factor VIIa may be useful to stop haemorrhage and to avoid surgery in patients not responsive to surgical therapy [6]. If rupture occurs and the patient is unstable haemodynamically, surgery can be necessary. Operative techniques and hemostatic mesh can be used for the bleeding surface of liver and the omentum can be sutured to the bleeding surface [10]. When the haemorrhage cannot be controlled with conservatively or with surgical techniques and acute liver failure occurs, liver transplantation should be considered [11].

Sibai reported a 13-year retrospective review of three patients with SLH. Two of them were managed conservatively and discharged from hospital. The other patient underwent hepatic resection and had mortality due to multiple organ failure [12]. In another study, Wicke et al. reported a review of 5 patients with subcapsular liver hematoma [13]. Three patients of them were managed conservatively and two required urgent surgical intervention, one of whom required liver transplantation.

In our case, TA-USG and CT showed subcapsular liver hematoma and free fluid in the abdominal cavity without hepatic rupture. Our case was haemodynamically stable, managed conservatively with blood products and steroids for treatment of HELLP syndrome, and followed up daily with imaging techniques.

In conclusion, the subcapsular liver hematoma with HELLP syndrome or/and severe preeclampsia is a rare clinical entity and should be suspected in signs of clinical symptoms. Close monitoring of these patients with HELLP syndrome by advanced imaging techniques in pre- and postpartum period is mandatory. If the patient's vital signs are stable, conservative management should be the first choice of treatment.
